# THE MARGRET M. AND PAUL B. BALTES AWARD LECTURE AND PRESENTATION

**DOI:** 10.1093/geroni/igad104.0533

**Published:** 2023-12-21

**Authors:** Shannon Jarrott

## Abstract

The Margret M. and Paul B. Baltes Award Lecture will feature an address by the 2022 Baltes Award recipient, Eric S. Kim, PhD, of the University of British Columbia. This session will also include the presentation of the 2023 Margret M. and Paul B. Baltes Award to recipient Reuben Ng, PhD of the National University of Singapore. The Margret M. and Paul B. Baltes Foundation Award in Behavioral and Social Gerontology recognizes outstanding early-career contributions in behavioral and social gerontology. The award is generously funded by the Margret M. and Paul B. Baltes Foundation.

